# Association of antenatal care with facility delivery and perinatal survival – a population-based study in Bangladesh

**DOI:** 10.1186/1471-2393-12-111

**Published:** 2012-10-16

**Authors:** Jesmin Pervin, Allisyn Moran, Monjur Rahman, Abdur Razzaque, Lynn Sibley, Peter K Streatfield, Laura J Reichenbach, Marge Koblinsky, Daniel Hruschka, Anisur Rahman

**Affiliations:** 1 Centre for Reproductive Health, icddr,b, Mohakhali, Dhaka, 1212, Bangladesh; 2 Emory University, 1520 Clifton Road, NE, Atlanta, GA, 30322-4027, USA; 3 John Snow Inc, 1776 Massachusetts Avenue, Suite 300, Washington, DC, USA; 4 School of Human Evolution and Social Change, Arizona State University, P.O. Box 872402, Tempe, AZ, 85287 – 2402, USA

## Abstract

**Background:**

Antenatal Care (ANC) during pregnancy can play an important role in the uptake of evidence-based services vital to the health of women and their infants. Studies report positive effects of ANC on use of facility-based delivery and perinatal mortality. However, most existing studies are limited to cross-sectional surveys with long recall periods, and generally do not include population-based samples.

**Methods:**

This study was conducted within the Health and Demographic Surveillance System (HDSS) of the International Centre for Diarrhoeal Disease Research, Bangladesh (icddr,b) in Matlab, Bangladesh. The HDSS area is divided into an icddr,b service area (SA) where women and children receive care from icddr,b health facilities, and a government SA where people receive care from government facilities. In 2007, a new Maternal, Neonatal, and Child Health (MNCH) program was initiated in the icddr,b SA that strengthened the ongoing maternal and child health services including ANC. We estimated the association of ANC with facility delivery and perinatal mortality using prospectively collected data from 2005 to 2009. Using a before-after study design, we also determined the role of ANC services on reduction of perinatal mortality between the periods before (2005 – 2006) and after (2008–2009) implementation of the MNCH program.

**Results:**

Antenatal care visits were associated with increased facility-based delivery in the icddr,b and government SAs. In the icddr,b SA, the adjusted odds of perinatal mortality was about 2-times higher (odds ratio (OR) 1.91; 95% confidence intervals (CI): 1.50, 2.42) among women who received ≤1 ANC compared to women who received ≥3 ANC visits. No such association was observed in the government SA. Controlling for ANC visits substantially reduced the observed effect of the intervention on perinatal mortality (OR 0.64; 95% CI: 0.52, 0.78) to non-significance (OR 0.81; 95% CI: 0.65, 1.01), when comparing cohorts before and after the MNCH program initiation (Sobel test of mediation *P* < 0.001).

**Conclusions:**

ANC visits are associated with increased uptake of facility-based delivery and improved perinatal survival in the icddr,b SA. Further testing of the icddr,b approach to simultaneously improving quality of ANC and facility delivery care is needed in the existing health system in Bangladesh and in other low-income countries to maximize health benefits to mothers and newborns.

## Background

Antenatal Care (ANC) in pregnancy can play an important role in the uptake of evidence-based care vital to the health of women and their infants [1,2]. Key components of ANC include the communication of health-related information, screening for risk factors, the prevention and management of complications, and preparation for delivery in a safe place by skilled attendants [3-5]. Specific components which can significantly reduce maternal and infant mortality include tetanus toxoid immunization, iron supplementation, early detection and treatment of pre-eclampsia, preparation for transportation to a delivery site, and safe delivery education among others [6,7]. The ultimate aim of ANC is to produce healthy babies and healthy mothers at the end of pregnancy [1].

A number of studies have assessed the effectiveness of ANC on both the uptake of safe delivery care and the reduction of maternal and infant mortality [8]. Studies report that ANC visits are associated with skilled delivery care. However, these studies are cross-sectional in design [9,10], and are based on a selected population [9]. Evaluations of ANC in different settings around the world, including Bangladesh, find a protective effect of ANC usage on low-birth-weight and preterm birth [11-15]. A few other studies do not find such associations [16,17]. These studies have specific limitations including that they are cross-sectional [11-13,15] or retrospective [14], and none address the issue of gestational age at birth which may cause selection bias due to the probability of low uptake of ANC among women who deliver prematurely. Prior studies have also reported a protective effect of ANC on perinatal mortality in both developing and developed countries [14,18,19], with one observing a dose-dependent association between the number of ANC visits and adverse pregnancy outcomes [19].

Despite these promising results, findings from previous studies have several limitations. Many studies rely on data collected with long recall periods, lack information regarding specific components in the ANC package, and do not include important covariates for adjustment. Importantly, there are few population-based studies of the effect of ANC on perinatal mortality, especially in low-income countries. This paper addresses some of these concerns by analyzing prospective data from a Health and Demographic Surveillance System (HDSS) in two areas offering separate ANC service delivery systems. Using this data, the paper assessed the association between the number of ANC visits and two outcomes—uptake of facility delivery care and perinatal mortality. It also determined the degree to which increased uptake of ANC could account for the reduction of perinatal mortality observed in a recently completed integrated Maternal, Neonatal and Child Health (MNCH) program in rural Matlab, Bangladesh [20].

## Methods

### Study site

This study was conducted in Matlab - a rural sub-district in Chandpur district situated 55 kilometers southeast of the capital Dhaka, Bangladesh. International Centre for Diarrhoeal Disease Research, Bangladesh (icddr,b) has been operating the HDSS in a population of about 220,000 since 1966 in the area. In the HDSS area, Community Health Research Workers (CHRWs) collect vital events such as marriage, birth, death, migration (both out- and in) and selected morbidity data from women of reproductive age (15 – 49 years) and children less than five years of age through bimonthly household visits.

The present study was conducted in the HDSS area with two distinct service provision zones: the icddr,b service area (SA) and the government SA. In the icddr,b SA, women and children receive health care from providers employed by icddr,b. It is divided into 4 administrative blocks with each block covering a population of about 27,000. Each block provides 24-hour delivery care at a sub-center staffed by midwives. Clinical activities in the sub-center are supported by a hospital in Matlab Township (icddr,b Hospital) staffed by medical doctors and nurses that offer basic obstetric care. In the government SA, the residents receive care from government health care providers. The government area is also served by five union-level sub-centers (each covering a population of about 25,000) and these centers are linked with the Upazila (sub-district) Hospital, located in the Matlab Municipality.

In the icddr,b SA, a Maternal Child Health and Family Planning program was initiated in 1977. In 1987, a safe motherhood program was introduced to increase the coverage of home births by midwives posted at the sub-centers. The home-based services were continued until 1996. Since then women have been encouraged to use the facility (sub-centre or icddr,b Hospital) for delivery care. In 2001, home-based delivery care was stopped and a facility-based strategy was fully established. In 2007, a new Maternal, Neonatal, and Child Health (MNCH) Program was initiated to strengthen the ongoing maternal and child health interventions and to add new evidence-based interventions following the continuum from pregnancy through the post-partum period to further reduce the already low level of perinatal mortality (48/1000 births) in the icddr,b SA. This new program further intensified integration of the health-service delivery modes from community to facility level. The details of the intervention and implementation process of the MNCH program have been described elsewhere [20].

### Study design and participants

This study took advantage of the ongoing HDSS and the recently completed integrated MNCH program. We evaluated the association of the number of ANC visits with facility delivery and perinatal mortality using prospectively collected data by CHRWs at home visits in icddr,b and government SAs during the period 2005–2009 (occurring monthly until 2006, and bi-monthly during 2007–2009). Additionally, we used a before-after study design within the 2005 – 2009 cohort in the icddr,b area to evaluate the impact of ANC services on reduction of perinatal mortality between the periods before (2005 – 2006) and after (2008–2009) implementation of the MNCH program [20]. Out of 29,800 pregnant women identified in household visits (2005–2009), 26,041 women gave birth during the study period and were included in the analysis.

### ANC packages

In the icddr,b SA, ANC service was provided to pregnant women according to the World Health Organization (WHO) recommendation of 4 focused ANC visits to low-risk pregnant women [21]. However, some changes were made in the content of the ANC packages after the introduction of the MNCH program in 2007. As part of the MNCH program, we standardized the interventions by training all staff, developed guidelines for all activities, established refresher training and routinely assessed the quality of care using a standard checklist. Prior to the implementation of the MNCH program the timing of ANC visits was flexible. However, with the inception of the MNCH program, the timing was fixed at with visits occurring at gestation week (GW) 15–19, GW 24, GW 32 and GW 36. A pregnancy notification was completed by CHRWs for all pregnant women identified during routine household surveillance visits. The completed form was then given to MNCH project staff during bi-weekly sub-center meetings for immediate data entry at icddr,b Matlab. An automated system was developed to generate the schedule of ANC visits according to the reported last menstrual period (LMP) of the pregnant woman. The ANC visit schedules were then sent to respective CHRWs, sub-centers and icddr,b Hospital. A copy of the visit schedule was also included in a Take Action Card, educational material used to remind women of danger signs [22]. ANC was provided in each of the four sub-centers and icddr,b Hospital and included a physical examination, risk identification and management, counseling including birth preparedness, and an ultrasound assessment to determine gestational age. If any complication was detected at the sub-center level, the woman was referred to icddr,b Hospital according to the guidelines. In addition, the CHRWs provided home visits during pregnancy to reinforce counseling messages received during ANC. CHRWs helped each woman to select a support person to assist her during labor and delivery. During ANC visits, the pregnant woman’s support person was encouraged to participate in counseling sessions to know the danger signs of mother and newborn, and also to know his or her role and responsibility to help the mother and infant during pregnancy, delivery and post-partum periods. In the Table 1, we describe the interventions provided in the icddr,b SA over the study period.

**Table 1:** Contents of antenatal care (ANC) package in icddr,b area, Matlab, Bangladesh

**Topics**	Activities* by ANC visits (1st visit: gestation week (GW) 15–19; 2nd visit: GW 24; 3rd visit: GW 32; 4th visit: GW 36)
**Rapport building**	*Establish rapport with pregnant women*
**History taking**	Current and past pregnancy, history of chronic disease, contraceptive use, and family history
**Clinical Examination**	
	*Maintain infection prevention measures (all visits)*
General	
	Height
	Weight
	Anemia
	Edema
	Blood pressure
Abdominal	
	Fundal height (2nd, 3rd and 4th visits)
	Fetal heart sound (2nd, 3rd and 4th visits)
	Fetal position and presentation (3rd and 4th visits)
Vaginal	(restricted unless physician decides)
Share findings	***Share the clinical findings with mother and support person***
**Ultrasound examination**	
	***Identify last menstrual period date (1st visit)***
	***Fetal growth (all visits)***
	***Early diagnosis of twin (1st visit)***
	***Any congenital malformation (1st and 2nd visit)***
	***Placental position (2nd , 3rd and 4th)***
	***Amount of liquor (2nd, 3rd and 4th)***
	***Mal-presentation (3rd and 4th)***
**Routine Investigations**	
	Urine dip-stick – albumin and sugar
	*Routine and microscopic examination (icddr,b Hospital)*
	*Blood grouping and hemoglobin estimation (icddr,b Hospital)*
**Immunization and drugs**	
	Immunization against tetanus
	***Anti- helminth drug use (2nd visit)***
	Iron and folic acid supplementation and ***checking***
**Risk factor identification and management**	
	***Antibiotic use for pre-mature rupture of membrane***
	***Corticosteroid treatment for women with risk or in preterm labor***
	***Induction of labor for postdated pregnancy***
	***Asymptomatic bacteriuria (icddr,b Hospital)***
	***Anti-D for Rh-negative mother***
**Tracking**	
	***Tracking of women with risk factors***
**Counseling**	
	*Diet and rest including work sharing and hygiene practice*
	*Immunization*
	*Micronutrient supplementation*
	***Birth preparedness (money, transport, pieces of cloths, delivery place)***
	*Breast feeding*
	***Preterm and/or low birth weight***
	***Immediate newborn care***
	*Danger signs of newborn and mother*
**Documentation**	*Proper documentation of all services*

*The items belong to column heading ‘Activities’ are presented by font-weight indicating the changes of interventions over the study period: normal - interventions unchanged; Italic: interventions strengthened; bold and italic: new interventions added.

In the government SA, there was also a policy of providing at least 4 ANC visits to pregnant women based on WHO recommendations. The contents of the ANC package in the government SA were supposed to cover history taking, measurement of weight and blood pressure including laboratory testing for selected morbidities, risk assessment and educating women regarding danger signs including counseling on evidence-based practices as illustrated in the Table 1. However the interventions in the ANC package provided through government health facilities were not consistently provided resulting in considerable variation in quality and uptake of ANC services [23].

### Data collection

Pregnant women were identified during regular CHRW home visits. Women who reported missing their LMP were given a urine test to verify pregnancy. Identified pregnant women were then followed routinely for data collection. Information on ANC visits including place of service and provider were collected in both the icddr,b SA and government SA. Stillbirth was defined as birth of a dead fetus after 28 weeks of gestation and live birth as birth of a fetus with any sign of life and early neonatal death as death of a live-born baby within 7 days. Perinatal death was defined as fetal (stillbirth) or early neonatal death. For each pregnant woman, delivery information such as place and type of delivery was also collected.

Detailed information on women’s age, parity, education, and household assets were collected from the HDSS databases and confirmed during interviews with study participants. Information on date of delivery was also retrieved from the HDSS database. Parity was defined as number of live or dead children before the current pregnancy. Educational status was assessed as number of completed years of school. Economic status was assessed by generating scores through principal-components analysis based on household assets of ownership of a number of consumer items (radio, watch, etc.), dwelling characteristics (wall and roof material), and type of drinking water and toilet facilities. These scores were then indexed into quintiles, where 1 represented the poorest and 5 the richest [24]. Last menstrual period date was determined by recall during the pregnancy-identification interview at the household visit. Gestational age at pregnancy outcome was measured by subtracting the LMP date from date of pregnancy outcome and was expressed in weeks.

The HDSS data were collected by CHRWs who have more than 10 years of schooling and are residents of the study site. CHRWs received a one-month intensive training on recruitment to orient them to the data collection tools. Refresher trainings were built into the program. Quality of data is checked carefully in the field and at the data entry level through random field visits by supervisors. In addition, a quality control team repeats a sub-sample of data collected at the field level for consistency checking.

### Statistical analysis

The associations of ANC visits with outcomes (delivery place and perinatal mortality) were assessed in icddr,b and government SAs with a logistic regression model. Results of logistic regressions were presented as odds ratios (ORs) with their 95% confidence intervals (CIs). In the analysis, antenatal care was grouped by number of ANC visits: 0 or 1 visit, 2 visits and ≥ 3 visits. Place of delivery was divided into home delivery or facility delivery.

Among the covariates maternal age was recorded into <20, 20 – 24, 25 – 34, ≥35 years, parity into 0, 1, ≥2, and education into 0, 1 – 5, ≥ 6 years of schooling. Socio-economic status was categorized by quintiles of asset scores. Gestational age was categorized into <37 or ≥37 GWs groups. Chi-square (χ^2^)-statistics were used to determine the association of background characteristics including calendar year of birth with ANC visits and outcomes. Wald statistics were used to determine the significance when the analysis was performed in logistic regression model. All the covariates related with the outcomes of interest were included in the multivariate model to adjust for potential confounding. We also performed stratified analyses dividing the cohort into 2005 – 2006 and 2008–2009 to observe whether the effect of ANC on perinatal mortality remained consistent over two periods in the icddr,b SA.

To determine how increasing ANC visits in icddr,b SA accounted for observed changes in perinatal mortality during the MNCH program period, we used logistic regression and assessed the change in magnitude and significance of the pre-post intervention dummy variable (before = 0; after =1) when adjusting for delivery place, gestational age, and ANC visits consecutively in different models. To assess whether ANC visits significantly mediated the effect of the MNCH intervention on perinatal mortality, we conducted a Sobel test. A Sobel test of mediation assesses whether the reduction of an independent variable’s effect (e.g. post-intervention) on a dependent variable (e.g. perinatal morality), after including the mediator in the model (e.g. number of antenatal care visits), is a statistically significant reduction [25].

### Ethical considerations

All women from icddr,b SA gave written consent for participation in the MNCH study. In addition we also used routine data collected by the ongoing HDSS. This study was approved by the Research and Ethical Review Committees of International Centre for Diarrhoeal Disease Research, Bangladesh.

## Results

### Sample description

Out of 26,041 pregnant women included in the study, 13,291 women were from the icddr,b SA and 12,750 women from the government SA. The background characteristics were significantly different between the two areas and are presented in Table 2. Women in the icddr,b SA were more educated, had fewer children, and were more likely to deliver by cesarean section compared to women from the government SA.

**Table 2:** Characteristics of women participating in the study in Matlab, Bangladesh 2005-2009

Variable name*	**icddr,b service area**	**Government service area**	***P*-value†**
	**No. (%)**	**No. (%)**	
**Age in year**			
<20	1865 (14.0)	1517 (11.9)	<0.001
20 - 24	4198(31.6)	3955(31.0)	
25 - 34	5945 (44.7)	5977 (46.9)	
≥ 35	1283 (9.7)	1301(10.2)	
Mean (± SD) age in year	26.3 (±5.9)	26.6 (±5.83)	
**Parity**			
0	5048 (38.0)	4514 (35.4)	<0.001
1 -2	6423 (48.3)	5872 (46.1)	
≥3	1820 (13.7)	2364 ( 18.5)	
Median	1	1	
**Education in year**			
0	2403(18.1)	2713 (21.3)	<0.001
1 – 5	3387 (25.5)	4080 (32.0)	
≥ 6	7501 (56.4)	5957 ( 46.7)	
Median	6	5	
**Asset quintiles**			
1-poorest	2043 (15.4)	2058 (16.1)	<0.001
2	2277 (17.1)	2236 (17.5)	
3	2711 (20.4)	2980 (23.4)	
4	3006 (22.6)	2786 (21.9)	
5-richest	3254 (24.5)	2690 (21.1)	
**Gestational age (GA) in weeks**			
<37	1979 (14.9)	2063 (16.2)	0.004
≥ 37	11312 (85.1)	10687 (83.8)	
Median GA in weeks	39	39	
**Delivery type**			
Vaginal	11714 (88.2)	11745 (92.2)	<0.001
Cesarean	1569 (11.8)	994 (7.8)	

SD: standard deviation.

* all are presented in percentages unless otherwise mentioned.

† *χ*^*2*^-test.

### Antenatal care, facility delivery, and perinatal mortality

**Figure 1:**
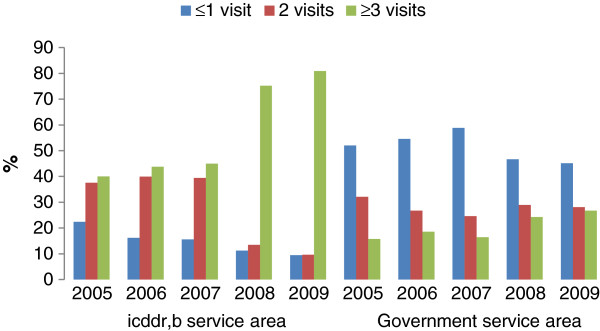
Antenatal care visits by year in the study area Matlab, Bangladesh 2005–2009.

There is wide variation in ANC coverage between icddr,b and government SAs (Figure 1). The proportion of women with 3 or more ANC visits increased from 40% to 81% in the icddr,b SA during the period of observation, while in the government SA the increase was from 16% to 27%. The calendar year of birth was associated with facility delivery in icddr,b and government SAs; however, it was associated with perinatal mortality only in the icddr,b area (Table 3). The facility delivery rate increased significantly in both areas: the rate increased from 51% to 77% and from 13% to 25%, respectively, in icddr,b and government SAs during the observation period (Table 3). The perinatal mortality rate reduced significantly in the icddr,b SA (from 4.8% to 2.9%) during the study period. We did not observe any decrease in perinatal mortality in the government SA (Table 3).

**Table 3:** Change in place of delivery and perinatal mortality in study area Matlab, Bangladesh 2005 - 2009

**Year**	**Place of delivery**				**Perinatal mortality**	
	icddr,b SA*		Government SA*		icddr,b SA*	**Government SA†**
	**Facility (%)**	**Home (%)**	**Facility (%)**	**Home (%)**	**Rate/100 births**	**Rate/100 births**
2005	51	49	13	87	4.8	5.9
2006	58	42	14	86	4.8	5.8
2007	58	42	18	82	4.1	6.1
2008	68	32	19	81	3.4	5.5
2009	77	23	25	75	2.9	5.7

SA: service area.

*P<0.05 (*χ*^*2*^-test).

†P>0.05 (*χ*^*2*^-test).

### Bivariate associations of antenatal care visits and perinatal mortality

In bivariate analysis, a number of socio-demographic and maternal factors were associated with ANC visits in both areas (Table 4). We observed associations of perinatal mortality with all available background indicators except asset index and type of delivery in both areas (Table 5). We also observed similar association of available covariates with facility delivery (data not shown).

**Table 4:** Association between background characteristics and antenatal care (ANC) visits in study area Matlab, Bangladesh 2005-2009

**Variables**	**icddr,b service area**				**Government service area**			
	**0 -1 ANC**	**2 ANC**	**≥ 3 ANC**		**0 -1 ANC**	**2 ANC**	**≥ 3 ANC**	
	**n (%)**	**n (%)**	**n (%)**	*P*-value*	**n (%)**	**n (%)**	**n (%)**	*P*-value*
**Age in year**								
<20	269 (14.4)	539 (28.9)	1057 (56.7)	<0.001	640 (42.2)	502 (33.1)	375 (24.7)	<0.001
20 - 24	572 (13.6)	1180 (28.1)	2446 (58.3)		1862 (47.1)	1226 (31.0)	867 (21.9)	
25 - 34	889 (15.0)	1644 (27.7)	3412 (57.4)		3290 (55.0)	1550 (25.9)	1137 (19.0)	
≥ 35	267 (20.8)	367 (28.6)	649 (50.6)		797 (61.3)	311 (23.9)	193 (14.8)	
**Parity**								
Null parity	647 (12.8)	1462 (29.0)	2939 ( 58.2)	<0.001	1829 (40.5)	1484 (32.9)	1201 ( 26.6)	<0.001
1 -2	956 (14.9)	1742 (27.1)	3725 (58.0)		3176 (54.1)	1606 (27.4)	1090 (18.6)	
3 or more	394 (21.6)	526 (28.9)	900 (49.5)		1584 (67.0)	499 (21.1)	281 (11.9)	
**Education in year**								
0	459 (19.1)	690 ( 28.7)	1254 (52.2)	<0.001	1544 (56.9)	713 ( 26.3)	456 (16.8)	<0.001
1 – 5	524 (15.5)	944 (27.9)	1919 (56.7)		2372 (58.1)	1092 (26.8)	616 (15.1)	
≥ 6	1014 (13.5)	2096 (27.9)	4391 (58.5)		2673 (44.9)	1784 (29.9)	1500 (25.2)	
**Asset quintiles**								
1-poorest	387 (18.9)	574 (28.1)	1082 (53.0)	<0.001	1322 (64.2)	494 (24.0)	242 (11.8)	<0.001
2	349 (15.3)	681 (29.9)	1247 (54.8)		1333 (59.6)	574 (25.7)	329 (14.7)	
3	444 (16.4)	780 (28.8)	1487 (54.9)		1577 (52.9)	850 (28.5)	553 (18.6)	
4	397 (13.2)	821 (27.3)	1788 (59.5)		1295 (46.5)	849 (30.5)	642 (23.0)	
5-richest	420 (12.9)	874 (26.9)	1960 (60.2)		1062 (39.5)	822 (30.6)	806 (30.0)	
**Gestational age in weeks**								
<37	497 (25.1)	664 (33.6)	818 (41.3)	<0.001	1178 (57.1)	551 (26.7)	334 (16.2)	<0.001
≥ 37	1500 (13.3)	3066 (27.1)	6746 (59.6)		5411 (50.6)	3038 (28.4)	2238 (20.9)	
**Delivery type**								
Vaginal	1792 (15.3)	3353 (28.6)	6569 (56.1)	<0.001	6281 (53.5)	3289 (28.0)	2175 (18.5)	<0.001
Cesarean section	205 (13.1)	371 (23.6)	993 (63.3)		302 (30.4)	299 (30.1)	393 (39.5)	

* *χ*^*2*^-test.

**Table 5:** Association between background characteristics and perinatal mortality among women in Matlab, Bangladesh 2005–2009

**Variables**	**icddr,b service area**		**Government service area**	
	**No. of perinatal death (%)**	P-value*	**No. of perinatal death (%)**	P-value*
**Age in year**				
<20	65 (3.5)	<0.001	74 (4.9)	<0.001
20 - 24	137 (3.3)		211 (5.3)	
25 - 34	253 (4.3)		347 (5.8)	
≥ 35	78 (6.1)		107 (8.2)	
**Parity**				
0	274 (5.4)	<0.001	377 (8.4)	<0.001
1 -2	194 (3.0)		235 (4.0)	
>=3	65 (3.6)		127 (5.4)	
**Education in year**				
0	132 (5.5)	<0.001	191 (7)	0.002
1 – 5	128 (3.8)		241 (5.9)	
≥ 6	273 (3.6)		307 (5.2)	
**Asset quintiles**				
1- poorest	97 (4.7)	0.274	139 (6.8)	0.100
2	83 (3.6)		120 (5.4)	
3	115 (4.2)		187 (6.3)	
4	119 (4.0)		154 (5.5)	
5- richest	119 (3.7)		139 (5.2)	
**Gestational age in weeks**				
<37	220 (11.1)	<0.001	273 (13.2)	<0.001
≥ 37	312 (2.8)		466 (4.4)	
**Delivery type**				
Vaginal	472 (4.0)	0.579	682 (6.8)	0.627
Cesarean section	58 (3.70)		54 (5.4)	

* *χ*^*2*^-test.

### Relationship between antenatal care visits and facility delivery

We observed significant association between ANC visits and facility delivery in the icddr,b and government SAs. The adjusted odds ratios of facility delivery among women attending 3 or more ANC visits in comparison to women with 0 or 1 ANC were 3.25 (95% CI: 2.63, 3.91) and 2.74 (95% CI: 2.43, 3.09), respectively, in icddr,b and government SAs. Significant dose-dependent associations were also observed (*P*-*for linear trend* <0.05) (Table 6).

**Table 6:** Association between antenatal care (ANC) and facility delivery among pregnant women in Matlab, Bangladesh 2005-2009

	**No. of ANC**	**Unadjusted odds ratio (95% confidence interval)**	Adjusted odds ratio (95% confidence interval)*
icddr,b service area‡			
	0 or 1†	1	1
	2	1.93 (1.72, 2.15)	1.92 (1.71, 2.16)
	≥3	3.87 (3.49, 4.29)	3.25 (2.63, 3.91)
Government service area‡			
	0 or 1†	1	1
	2	2.06 (2.09, 2.55)	1.71 ( 1.52, 1.92)
	≥3	3.91 (3.49, 4.38)	2.74 (2.43, 3.09)

*adjusted for maternal age, parity, education, asset quintiles, gestational age and calendar year of birth.

† reference category.

‡*P-for linear trend* <0.05.

### Relationship between antenatal care visits and perinatal mortality

We observed an association between ANC visits and perinatal mortality in the icddr,b SA but not in the government SA (Table 7). In the icddr,b SA, the adjusted odds of perinatal mortality was about 2-times higher (OR 1.91; 95% CI: 1.50, 2.42) among women who received ≤1 ANC compared to women who received ≥3 ANC visits. We also observed a significant dose-dependent association between number of ANC visits and perinatal mortality in the icddr,b SA (*P-for linear trend <0.05*) (Table 7). In the stratified analysis, we observed similar associations of ANC in both time periods before and after the MNCH program. The adjusted odds ratios of perinatal mortality among women who received 0 or 1 ANC in comparison to women with 3 or more ANC visits were 1.97 (95% CI: 1.41, 2.77) and 1.92 (95% CI: 1.56, 3.44), respectively, before and after implementation of the MNCH program.

**Table 7:** Association between antenatal care (ANC) and perinatal mortality in Matlab, Bangladesh 2005-2009

	**No. of ANC**	**Unadjusted odds ratio (95% confidence interval)**	Adjusted odds ratio (95% confidence interval)*
icddr,b service area ‡			
	0 or 1	2.48 (1.98, 3.10)	1.91 (1.50, 2.42)
	2	1.89 (1.55, 2.31)	1.54 (1.24, 1.90)
	≥3†	1	1
Government service area			
	0 or 1	0.99 (0.82,1.20)	1.04 (0.87, 1.24 )
	2	0.86 (0.69,1.06)	0.84 (0.67, 1.05)
	≥3†	1	1

*adjusted for maternal age, parity, education, asset quintiles, gestational age and calendar year of birth.

† reference category.

‡*P-for linear trend* <0.05.

### Role of antenatal care visits in reducing perinatal mortality in the icddr,b service area

A base model (adjusted with age, parity, education and asset score) predicting perinatal mortality in the icddr,b SA showed a 36% reduction in odds of perinatal mortality (OR 0.64; 95% CI: 0.52, 0.78) among women giving birth after the introduction of the MNCH program in comparison to women delivering before the program (Table 8) [20]. Adjusting for delivery place (home or facility) and gestational age did not change the effect estimates, and the reduction of perinatal mortality remained statistically significant. Adjusting for number of ANC visits attenuates the reduction in odds observed during the intervention (OR 0.81; 95% CI: 0.65, 1.01) and renders it statistically non-significant (Table 8). A Sobel test indicates that this reduction of effect when adjusting for ANC visits is statistically significant (*P* < 0.001) suggesting that increased ANC coverage accounted for a major portion of the reduction in odds of the perinatal mortality observed during the intervention. Moreover, this reduction in odds of perinatal mortality could not be accounted for by increased facility use for delivery or by aspects of the intervention that changed gestational age.

**Table 8:** Antenatal care (ANC) and perinatal mortality before and after Maternal, Neonatal, and Child Health (MNCH) program in icddr,b area Matlab, Bangladesh

	**Adjusted odds ratio (95% confidence intervals)Â¶**			
	Model 1*	**Model 2†**	Model 3**	**Model 4‡**
**Intervention period**				
Before (2005–2006)Â§	1	1	1	1
After (2008–2009)	0.64 (0.52, 0.78)	0.62 (0.51, 0.77)	0.67 (0.54, 0.82)	0.81 (0.65, 1.01)
**Delivery place**				
HomeÂ§		1	1	1
Facility		1.13 (0.91, 1.41)	1.18 (0.94, 1.47)	1.36 (1.08, 1.71)
**Gestational age (weeks)**				
<37			4.93 (4.00, 6.08)	4.40 (3.56, 5.43)
>=37Â§			1	1
**No. of ANC visits**				
0 or 1				2.41 (1.84, 3.16)
2				1.89 (1.48, 2.42)
≥3Â§				1

Â¶consecutive adjustment of covariates in the model and changes of odds ratios of perinatal mortality before (2005–2006) and after (2008–2009) the MNCH interventions in the icddr,b service area, Matlab, Bangladesh.

Â§ reference category.

*adjusted for maternal age, parity, education, and asset quintiles.

†adjusted for the covariates in model 1 + delivery place.

**adjusted for the covariates in model 2 + gestational age.

‡adjusted for the covariates in model 3 + no. of antenatal care visits.

## Discussion

In this study, women’s use of antenatal services increased considerably over the course of the 3-year MNCH intervention, with the number of women receiving 3 or more ANC visits increasing from 40% to 81%. In both areas (icddr,b and government SAs), women receiving more ANC visits were more likely to seek facility-based delivery care. However, increased ANC visits were only associated with reduced perinatal mortality in the icddr,b area. In addition, increased ANC visits appear to mediate a large portion of the observed 36% reduction in the odds of perinatal mortality during the MNCH intervention in icddr,b SA [20]. This was likely a direct result of the ANC provided rather than a subsidiary increase in use of skilled facility care, as increased facility delivery did not mediate the reduction in perinatal mortality. The study findings confirm earlier observations that ANC is associated with positive health care behavior and improved perinatal survival [6,26,27]. It also suggests that standardized antenatal care provided through the program may have played a substantial role in reducing perinatal mortality (particularly early neonatal mortality) - an important indicator in achieving Millennium Development Goal (MDG) for child survival (MDG-4) in low-income settings.

The present study has several strengths. Prospectively collected information on ANC visits and outcome data in two areas, one of which implemented an MNCH intervention aimed at improving ANC coverage and quality, permits us to examine the effect of a changing pattern of ANC use on perinatal survival. Important social and demographic factors including gestational age were also available for adjustment of potential confounding effects. However, we were unable to determine to what extent the individual intervention contributed to the perinatal mortality reduction. Also, we did not collect information on how ANC facilitated the referral of complicated women and neonates from community to facility level in the government SA. This information (referral and subsequent treatment) was collected in icddr,b SA but is not currently available and will be reported separately.

There are several study limitations which need to be acknowledged. Due to the long-standing acceptability and proven effectiveness of several interventions included in the ANC program, randomization is not a feasible option for the package; as a result, observational studies are a necessary design [1]. Due to differences of ANC packages between icddr,b and government SAs, it is difficult to compare the effect of ANC across the two sites. However, prior to the strengthening of ANC services by the MNCH program when the components of ANC services were more comparable between two areas, the effect of ANC was only observed in the icddr,b SA. The observed lack of association between ANC and perinatal mortality in the government SA suggests the existing ANC service in the government SA is not providing the expected benefits due to lack of quality of care and linkage between community and facility-based services. In the second analysis we used a before-after design to examine further how the use of ANC services after initiation of the MNCH program played a role in the observed reduction of perinatal mortality. We describe in detail the limitations, especially the difficulty of comparing across service areas and the challenge of inferring the intervention effect with observational data [20]. In the present study, we adjusted for important socio-demographic variables including the calendar of year of birth to address potential confounding. Three findings, (i) the observed high effect estimates (odds ratio about 2), (ii) the dose–response relationship between exposure to ANC and outcome (perinatal mortality), and (iii) the apparent mediation of the intervention effect by high ANC coverage, provides additional support for the effectiveness of the ANC services in the present study. Nonetheless, the associations observed may be due to factors related to the selection of study groups or to other forms of unmeasured confounding.

ANC visits were strongly associated, in a dose-dependent way, with perinatal mortality in the icddr,b SA, but we did not observe such effects in the government SA. ANC visits may improve perinatal outcome for several reasons. First, ANC introduces women and their families to the formal health system which makes them more likely to use health facilities for delivery [27-29]. The fact that increased facility use for delivery did not mediate the post-intervention reduction in perinatal mortality makes this hypothesis less plausible. Second, antenatal education (counseling) can increase the understanding of mothers and/or family members about early recognition of danger signs during pregnancy and delivery and basic newborn care, and therefore facilitates the use of the existing health system or improved home care (such as warmth, exclusive/immediate breast feeding) at appropriate times [27,30,31]. Third, incorporating and maintaining quality evidence-based interventions in the ANC package may also increase the coverage of services conducive to improving perinatal health. The availability of evidence-based interventions and the presence of an effective linkage between community and facility-based services might explain the observed difference of associations between ANC visits and perinatal mortality in two areas.

Another important finding of the present study is that achieving high ANC coverage in the icddr,b SA explained much of the 36% reduction of perinatal mortality observed due to initiation of MNCH program. Among potential mediators of this reduction, including facility use, gestational age, and increased number of antenatal visits, the number of ANC visits was the only factor that substantially attenuated the observed post-intervention reduction in perinatal mortality (Table 8). This finding suggests that ANC services played a key role in observed perinatal mortality reduction in the icddr,b area. In addition to a considerable increase in ANC coverage (women with ≥3 visits increased from 40% to 81%), the MNCH ANC package included several new components which were strengthened through training, refresher training, and regular quality control check-up of available services (Table 1). Counseling and training during each ANC session focusing on evidence-based practices, identifying risk factors, and involving support persons may have played an important role. Prenatal interventions such as birth preparedness, micronutrient and/or iron-folate and anthelmintic supplementation, risk tracking and appropriate management such as antibiotic use for preterm premature rupture of membrane, corticosteroid treatment for women with risk or in preterm labor, and induction of labor for post-term pregnancy are also recognized to improve perinatal health [6,32,33]. Although this study cannot discriminate the independent effects of these specific components, facilitating access to this suite of evidence-based interventions in the icddr,b area appears to have played an important part in improving perinatal survival. However, selection of an individual intervention should be based on the cost-effectiveness of the available evidence-based interventions.

Universal coverage of delivery care by skilled attendant is one target of MDG-5 [34]. The latest maternal health survey in Bangladesh shows that the national facility delivery rate increased significantly in the last 5 years to 23%, which is similar to the government SA in the present study [35]. In the present study we observed that ANC was associated with increased facility delivery practice in icddr,b and government SAs. Several studies in Bangladesh also report associations between ANC and increased uptake of facility delivery [4,36]. Our findings are also consistent with studies conducted in low- and middle-income countries that ANC uptake is associated with 3–4 times increase in facility delivery rate [28,37].

Very few studies have evaluated the association between ANC care and perinatal mortality in low- and middle-income countries. One study conducted in Kenya found that women who received two antenatal care visits had better perinatal outcomes than those who received less than two antenatal visits [11]. Another study in Jamaica found that mothers who started ANC in the first trimester decreased the risk of perinatal death [38]. It is difficult to compare the effect estimate observed in the present study with these earlier studies due to differences in services included in the studies. However, the observed 1.5 to 2 times increased risk of perinatal mortality among women with no ANC visits in earlier studies is similar to the findings we observed [11,39].

The feasibility of scaling up ANC services like those implemented in the icddr,b SA into the existing health system and program in Bangladesh or other low-income countries is an important and outstanding question. There is strong similarity between icddr,b SA and government SAs on the basic infrastructure and human resources. In Bangladesh, existing ANC care in government areas is provided by paramedics at union level sub-centers which are equivalent to the icddr,b sub-centers. In the sub-district level hospital, which is equivalent to icddr,b Matlab Hospital, ANC services are provided by nurse-midwives or doctors as is the case in the icddr,b hospital. The Government of Bangladesh has recently established about 16,000 community clinics, each covering a population of about 6,000, where basic ANC will be provided through community health workers. However, the success of ANC in the icddr,b area likely relied on several additional elements not currently available in the government SA: (1) guidelines for specific elements of the ANC package, (2), foundational and refresher training of health care providers including development of a users’ guide, (3) standardized counseling on evidence-based interventions and risk factor, and involving support person in this process, (4) linkages between outreach, community, and facility levels to improve the management of complications, and (5) a process of monitoring, evaluation and supervision of the offered services at regular intervals. In addition, a system needs to be put in place in the government SA to ensure appropriate supplies to meet local needs. These initiatives could be linked with other ongoing activities in many Upazilas (sub-districts) through the government services such as Demand Side Financing [40], which provides incentives for uptake of at least 3 ANC services for the poorest group of women in the community.

## Conclusions

The present study highlights the relationship of ANC with safe delivery practices and perinatal survival after adjustment for possible confounding factors. We report that high ANC coverage is associated with enhanced use of facility based delivery and increased perinatal survival in the icddr,b area where interventions within the ANC package are more focused, linkage between community- and facility-based services is established, and quality of services offered by health care providers is maintained through regular monitoring and training activities. In addition, much of the effect of the new MNCH intervention was explained by high coverage of ANC visits. As reflected from analysis of data from the government SA, the study also indicates that the ongoing ANC activities in Bangladesh might have little impact on improving perinatal survival even though ANC visits are found to increase the use of increased facility based delivery. The findings presented in this article suggest that ANC visits increase facility delivery and decrease perinatal mortality, two important indicators of maternal and child health. However, further testing of the icddr,b model is needed in the existing health system in Bangladesh and other low-income countries with simultaneous efforts to improve the quality of ANC care to maximize health benefits to mothers and newborns

## Abbreviations

ANC: Antenatal care; CHRWs: Community health research workers; CI: Confidence interval; HBLSS: Home based life saving skills; HDSS: Health and demographic surveillance system; icddr,b: International centre for diarrhoeal disease research, Bangladesh; OR: Odds ratio; MDG: Millennium development goal; MNCH: Maternal, neonatal and child health; SA: Service area.

## Competing interest

The authors declare that they have no competing interests.

## Authors’ contributions

JP, AM, LS, MK, DH, and AR contributed to the study concept and design. AR, JP, and AM supervised implementation of the study. JP, MR, AR, DH and AR coordinated collection of field data and contributed initial data cleaning and analyses. JP crafted the initial draft of the manuscript. All authors read and approved the final manuscript.

## Pre-publication history

The pre-publication history for this paper can be accessed here:

http://www.biomedcentral.com/1471-2393/12/111/prepub
